# Supplementary material for: A Liposome-Based Nanoparticle Vaccine Induces Effective Immunity Against EBV Infection
Source: Vaccines (Basel). 2025 Mar 28;13(4):360. doi: 10.3390/vaccines13040360 (PMC12031204; doi:10.3390/vaccines13040360)
Supplement: Supplementary file 1 [file vaccines-13-00360-s001.zip › vaccines-3453983-supplementary.pdf]

vaccines

MDPI

Supplementary Materials for:

A liposome-based nanoparticle vaccine induces effective immunity against EBV infection

Ping Li [1,2,#], Zihang Yu [3,#], Ziyi Jiang [1], Yike Jiang [2], Jingjing Shi [1], Sanyang Han [1,*] and Lan Ma [1,2,4,*]

1 Institute of Biopharmaceutical and Health Engineering, Tsinghua Shenzhen International Graduate School, Tsinghua University, Shenzhen, 518055, China; liping@szbl.ac.cn (P.L.), jiang-zy20@mails.tsinghua.edu.cn (Z.J.), shijj21@mails.tsinghua.edu.cn (J.S.).
2 Institute of Biomedical Health Technology and Engineering, Shenzhen Bay Laboratory, Shenzhen, 518132, China. jiangyk@szbl.ac.cn (Y.J.).
3 Institute of Bio-Architecture and Bio-Interactions, Shenzhen Medical Academy of Research and Translation, Shenzhen, 518107, China. yuzihang@smart.org.cn (Z.Y.).
4 State Key Laboratory of Chemical Oncogenomics, Tsinghua Shenzhen International Graduate School, Tsinghua University, Shenzhen 518055, China.
# These authors contributed equally to this work.
* Correspondence: hansanyang@sz.tsinghua.edu.cn (S.H.), malan@sz.tsinghua.edu.cn (L.M.)

Supporting Information Text

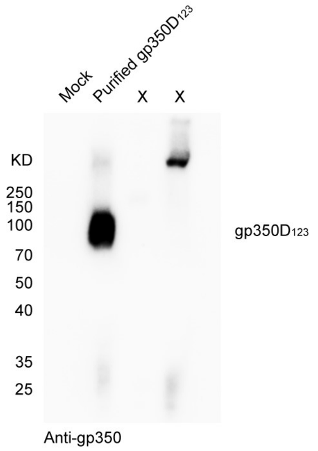

Figure S1. Western-blot detecting the purification of gp350D_{123} protein with gp350-specific antibody.

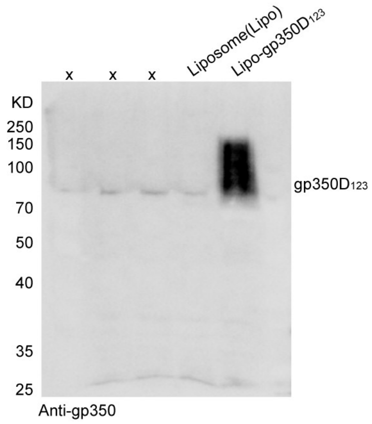

Figure S2. Western-blot detecting gp350D_{123} protein in Lipo-gp350D_{123} nanoparticles with gp350-specific antibody.
